# Record linked retrospective cohort study of 4.6 million people exploring ethnic variations in disease: myocardial infarction in South Asians

**DOI:** 10.1186/1471-2458-7-142

**Published:** 2007-07-05

**Authors:** CM Fischbacher, R Bhopal, C Povey, M Steiner, J Chalmers, G Mueller, J Jamieson, D Knowles

**Affiliations:** 1 Information Services Division, NHS National Services Scotland, Gyle Square, 1 South Gyle Crescent, Edinburgh EH12 9EB, UK; 2 General Register Office for Scotland, Ladywell House, Ladywell Road, Edinburgh EH12 7TF, UK; 3 Public Health Sciences, University of Edinburgh, Teviot Place, Edinburgh EH8 9AG, UK

## Abstract

**Background:**

Law and policy in several countries require health services to demonstrate that they are promoting racial/ethnic equality. However, suitable and accurate data are usually not available. We demonstrated, using acute myocardial infarction, that linkage techniques can be ethical and potentially useful for this purpose.

**Methods:**

The linkage was based on probability matching. Encryption of a unique national health identifier (the Community Health Index (CHI)) ensured that information about health status and census-based ethnicity could not be ascribed to an identified individual. We linked information on individual ethnic group from the 2001 Census to Scottish hospital discharge and mortality data.

**Results:**

Overall, 94% of the 4.9 million census records were matched to a CHI record with an estimated false positive rate of less than 0.1 %, with 84.9 – 87.6% of South Asians being successfully linked. Between April 2001 and December 2003 there were 126 first episodes of acute myocardial infarction (AMI) among South Asians and 30,978 among non-South Asians. The incidence rate ratio was 1.45 (95% CI 1.17, 1.78) for South Asian compared to non-South Asian men and 1.80 (95% CI 1.31, 2.48) for South Asian women. After adjustment for age, sex and any previous admission for diabetes the hazard ratio for death following AMI was 0.59 (95% CI 0.43, 0.81), reflecting better survival among South Asians.

**Conclusion:**

The technique met ethical, professional and legal concerns about the linkage of census and health data and is transferable internationally wherever the census (or population register) contains ethnic group or race data. The outcome is a retrospective cohort study. Our results point to increased incidence rather than increased case fatality in explaining high CHD mortality rate. The findings open up new methods for researchers and health planners.

## Background

Several countries require their services to show with accurate, quantitative data that they are meeting the needs of ethnic and racial minority populations. Such requirements for data are often impossible to implement [1]. The Race Relations (Amendment) Act 2000 and NHS policy, for example, require UK health services to demonstrate how successful they are in promoting racial equality and helping reduce ethnic inequalities. However, routine data sources in Scotland, as in most countries, rarely include a patient's ethnicity, and when this is available, it is not certain whether it is based on self-classification (as recommended) or on the view of a health care provider.

The 2001 Scotland Census, as in many countries including the USA, Canada, Australia and New Zealand [1], contains a question on ethnicity or race [2]. Although the census form may often be completed by one person on behalf of the household, it is likely that the answer to the census question is a good proxy for a person's own view of their ethnicity. We foresaw that if we could link the census records of individuals to the routinely collected health data on the same individuals, we could examine the relationship between ethnicity and disease for the population of Scotland.

The Information Services Division (ISD) of NHS National Services Scotland maintains a dataset of hospital episode data (derived from the Scottish Morbidity Record (SMR01)) which is linked internally to previous hospitalisations and death data (obtained from The General Register Office for Scotland). Thus a record can be built up for people that joins all their hospital admissions and includes their death where applicable. We aimed to link the census data to this SMR01-death linked dataset.

While it was conceptually feasible to link census data with morbidity and mortality records there were concerns about the legality of this proposal. Linkage between census data and mortality data has been performed before, with extensive experience in New Zealand [3]. There, Statistics New Zealand collect information on deaths and the census, and the two datasets have been matched [3]. Similarly, there is experience of such linkage in Scandinavia and other parts of Europe though, in the arena of ethnicity and health, it is less advanced [4]. Such work is usually based on a population register with an identification number that is also used in health records. Country of birth, but usually not ethnicity or race, is sometimes recorded. Cancer registry and mortality records within the England and Wales Longitudinal Study, which contains ethnicity from the census, have been linked prospectively to census data [5]. Within the Scottish Longitudinal Study there are also plans for linkage with hospital data, but it is only in 5% of the Scottish population, so may not be able to produce useful information by ethnic group [6].

This study advances the field by linking data from two agencies and four databases to explore ethnic variations in disease using the entire population of Scotland (about 5 million people). Under present legislation and guidance, including the data protection legislation, the Census Act 1920 and the Census Confidentiality Act 1991, the retrospective linkage described here would be acceptable if the confidentiality of individual personal information was maintained during the matching, handling and analysis of the matched dataset. To achieve this we used one-way encryption methods (otherwise known as "hashing") and organisational procedures. One-way encryption methods have been used to allow record linkage of anonymised data, but apparently not in the UK, and not for the purpose used here [3,7,8].

For this demonstration project we studied heart disease, since it is one of the most important conditions in Scotland, is common in ethnic minority groups, and its manifestations are likely to be recorded in hospital morbidity records. South Asians comprise the largest non-white minority ethnic group in Scotland [2]. Residents of England and Wales with ancestral origins in the Indian subcontinent (South Asians) have increased mortality from coronary heart disease (CHD) compared to most other ethnic minority groups, including people of European ancestry (White) [1,9,10]. The prevalence of CHD is also comparatively high, [11-14] but few data are available on incidence from population studies [15,16].

UK mortality data are based on country of birth, but the increase in the UK born ethnic minority population makes this an inaccurate guide to ethnic group variations, particularly in younger age groups [1,10]. Little information is available about CHD risk among South Asians living in Scotland, where CHD incidence and prevalence are higher than England and Wales. Our recent analysis of mortality by country of birth shows the expected excess in comparison to England and Wales, and modest excesses in relation to the population of Scotland [17].

For acute myocardial infarction (AMI), hospital discharge data combined with mortality data provide a good guide to incidence. However, in 2004/05, only around 6% of hospital discharges in Scotland included information on ethnic group. We created, using probability methods for record linkage, an anonymised dataset containing hospital discharges and deaths with a diagnosis of CHD in Scotland between 2001 and 2003 and ethnic group in the 2001 Census [17]. We describe, as an example of the potential of the dataset, the incidence of AMI and subsequent survival among South Asians in Scotland compared to the remaining population of Scotland (henceforth, non-South Asians). Appendix 1 summarises what was known before and now.

## Methods

### Procedural issues

The procedural techniques took into account that the required data are held by two different bodies, with different responsibilities. The community health index (CHI) and hospitalisation data are collected and maintained by ISD, and the census and mortality data are collected and maintained by the General Register Office for Scotland (GROS). GROS performed a risk-assessment of the data security aspects of the proposed record linkage and produced recommendations, including that all the linkage work was performed on a stand-alone computer. Modifications were made to the computer operating system to monitor activity and an audit trail was implemented for all peripheral devices (floppy and CD-ROM drives, and external ports). The computer was in a locked room at GROS accessed with the agreement of both organisations. GROS maintained a register of visits. Neither organisation could view the other organisation's primary datasets (the census data and the hospital discharge/death linked data) in a form in which individuals were identifiable.

### Use of record linkage and one-way encryption

The linkage methods used were identical to those developed in Scotland for administrative matching of health data. Date of birth, surname (using soundex codes to allow for variations in spelling), forename, address and full postcode were used to link. For many records there was an exact match. For the remaining records, a probability matching process was performed using methods pioneered by Newcombe [18]. Here, the rate of false positives is critical. Methods have been developed to identify how false positives occur and what kind of strategies a human checker employs to decide whether a pair match is good. These decision strategies were built into a partitioning computer algorithm, which groups (partitions) records according to the likelihood of correct matching. These partitions then allow the allocation of human checking to partitions, rather than individual records, so maximising linkage and minimising effort.

Prior to the linkage the project's steering group judged and specified that an effectiveness of 80% or more for all ethnic groups would be adequate for the purposes of this demonstration phase. This was a pragmatic decision that needed to be made (in the absence of data or theoretical precedent) to help assess the personnel time and resource to be directed to the matching process.

As both census and CHI datasets described essentially the same population, for every person on the CHI, there was a very high probability that the same individual was represented on the census. Where all linking variables matched, it was assumed that this represented the same individual. The various types of mismatches were addressed by the use of the partitioning algorithm described above. For example, all the instances of mismatch limited to one differing digit of year of birth were put into the same partition. A sample was then taken from each of the partitions, and the adequacy of each match was assessed by considering how much less likely the next best match was. This allowed quantification of the quality of the match for each partition.

**Figure 1:**
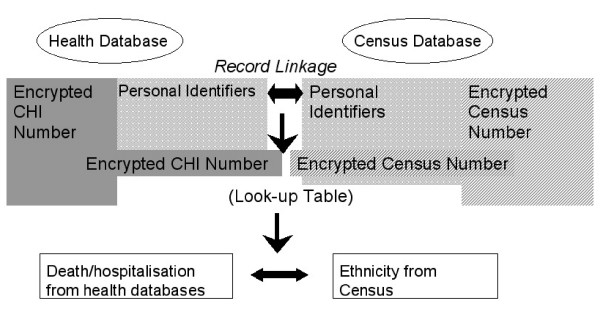
Anonymised linkage of health databases to census databases: conceptualising the procedure.

Figure 1 illustrates in concept how record linkage was based on the use of subsets from three datasets: the SMR01-death linked dataset, extracted by coronary heart disease diagnostic codes on any record, and including personal identifiers and clinical information; the CHI dataset which contains personal identifiers and the CHI number; and the census file which contains personal identifiers and details of individuals' ethnicity.

The CHI dataset lists everyone registered with a General Practitioner or in receipt of screening services and forms a unique identifier for NHS use. More than 99% of the Scottish population is listed. A CHI number was attached to the SMR01-death linked dataset. A one-way cryptographic (hashing) algorithm was then used to encrypt the CHI number into a code such that it is currently impossible to reverse the process. Personal identifiers were removed and dates of birth converted to ages to create a file suitable for analysis.

A census extract with personal identifiers and a unique census identification number was taken from GROS's census file. The census number was encrypted using an algorithm developed by GROS. The file was then linked to the encrypted CHI dataset using the probability methods already described. The personal identifiers were then removed, leaving a file with an encrypted CHI number and its corresponding encrypted census number.

A new census extract containing ethnic code and an encrypted census number was joined to the above file by exact matching using the encrypted census number. The encrypted census numbers were now discarded leaving the ethnicity code and the encrypted CHI number. The subset of the SMR01-death linked file was exactly linked via the encrypted CHI numbers; The encrypted CHI was replaced with an unrelated serial number (to keep the hospital spells together), resulting in depersonalised clinical health records carrying census ethnicity codes. To reduce the risk of disclosure some variables were further aggregated and access to the analysis file was restricted to named researchers in the secure room described above.

### Evaluation of the effectiveness of linkage between census data and CHI

The quality of the linkage depended on the adequacy of the linkage between the census data and the CHI. We tested the probability matching methods using data supplied by Scottish hospitals where the CHI number was present. We assessed whether the personal identifiers we used yielded the same CHI number.

### Ethical approval, legal advice and public scrutiny

As no identified individual responses to the Census were linked to health records, and vice versa, we were following the principles of existing data protection legislation and guidance, including two National Statistics (NS) Protocols on Data Access and Confidentiality, and Data Matching. Legal advice (obtained by GROS) confirmed that the proposed use of census data was compliant with the Census Act 1920 and the Data Protection Act 1998. We obtained approval from the Scottish Multi-centre Research Ethics Committee and the Scottish Privacy Advisory Committee (PAC).

This work was supported by the Commission for Racial Equality, NHS Health Scotland (through The National Resource Centre for Ethnic Minority Health), ISD, the Scottish Executive Health Department and the Chief Medical Officer. We disseminated information widely to encourage people to discuss the proposal and the early findings.

### Analysis of data on incidence of myocardial infarction and mortality

A diagnosis of AMI was recorded by a clinician at hospital discharge or recorded as the underlying cause of death on a death certificate. There is national guidance promoting uniformity of approach. The approach to the diagnosis would be similarly applied in different ethnic groups. Increasing use of laboratory tests makes the diagnosis less open to the subjective judgement of a clinician who might be influenced by the ethnicity of patients. The Scottish Morbidity Record database (SMR01) records inpatient and day case discharges with a diagnostic accuracy of around 90% for CHD [19]. Data on discharges with CHD diagnoses (ICD9 410–414 or ICD10 I20-I25 in any diagnostic position) between 1981 and December 2003 were extracted (the data from 1981 – 2001 being used to check for prior AMI). A first AMI was identified using ICD10 codes I21 and 122 and ICD9 code 410, but restricting to first admissions for AMI based on no hospital admission for AMI in the preceding 10 years. Deprivation categories were assigned using the postcode sector of residence in 2001, which provides the Scottish Index of Multiple Deprivation (SIMD). The SIMD scorers were divided into quintiles for analysis.

The South Asian population comprised people who were designated by the head of household at the 2001 census as Pakistani (23, 660 people, 86.9% matching of census and community health index), Indian (11, 690, 87.6% match), Bangladeshi (1463, 84.9% match), and other South Asian (4526, 82.2 % match).

The incidence of AMI was calculated using the data between April 2001 and December 2003 inclusive. Poisson regression modelling was used to calculate incidence rate ratios and 95% confidence intervals, by sex. Odds ratios for survival following AMI at fixed intervals from 30 to 720 days after admission were calculated using logistic regression. Kaplan-Meier plots were produced using an age adjusted survival function, setting age at 68 years. Adjusted hazard ratios were estimated using Cox regression. We included age (in five groups), sex and previous admission for diabetes in the Cox model. Graphical methods were used to confirm the proportional hazards assumption. Adjustment for area based social and economic deprivation based on the Scottish Index of Multiple Deprivation was done.

The checks included review of procedures and analysis programs created by M.S., first by C. F., and subsequently in consultation with an external adviser with experience of similar analyses, KMc (see acknowledgements).

## Results

### Quality of linkage of the subset of the SMR01-death file to CHI

630,121 SMR01-death records came to ISD with a CHI number applied by the hospitals supplying the data. 629,493 yielded the same CHI number on matching to the CHI database using the fields in our linkage methodology. 628 records had differing CHI numbers, and are therefore potential false linkages from our type of probability linkage. 125 of the 628 had CHI which do not appear in the CHI data set, so these must be errors on the original SMR01 record. 503 (628-125 entries) false positives might have arisen in the probability linkage process. Applying the worst assumption that all the 503 CHIs derived from the linkage were incorrect and the CHIs as applied by the hospitals were correct, this implies an upper limit to the false positive linkage rate of 0.08 %.

### Quality of overall linkage of census and CHI records

Table 1 shows that 94% of the 4.9 million census records were matched to a CHI record (with an estimated false positive rate of less than 0.1 %, as above). The proportion of matches exceeded 80% for every ethnic group, meeting our prior standard.

**Table 1:** Effectiveness of matching process by ethnic group

	**Unmatched**	**Matched**	**Total**	**% matched**
Any Mixed Background	1139	10429	11568	90.2%
Other Ethnic group	1446	7115	8561	83.1%
Non Resident Students	2887	33272	36159	92.0%
No Response	37768	180872	218640	82.7%
Scotland	293786	4605671	4899457	94.0%

White Scottish	201306	3890972	4092278	95.1%
Other White British	24976	321278	346254	92.8%
White Irish	4332	41078	45410	90.5%
Other White	9943	61279	71222	86.0%
Indian	1651	11690	13341	87.6%
Pakistani	3580	23660	27240	86.9%
Bangladeshi	261	1463	1724	84.9%
Other South Asian	980	4526	5506	82.2%
Chinese	2459	12161	14620	83.2%
Caribbean	196	1400	1596	87.7%
African	723	3598	4321	83.3%
Black Scottish or Other Black	139	878	1017	86.3%

### Myocardial infarction incidence and survival

Between the date of the Census (29th April 2001) and 31st December 2003 there were 126 first episodes of AMI among South Asians, of which 105 were admissions with AMI in the principal diagnostic position and 21 were sudden deaths in the community with an underlying cause of death of AMI. The corresponding figures for non-South Asians were 30,978 first episodes, 21,306 admissions and 9,672 community deaths. Table 2 shows that in most age groups the incidence of AMI was higher in South Asians than non-South Asians. Similar results were obtained when the analysis included both first and subsequent admissions for AMI; the directly standardised rate (95% *CI*) was 9.88 (7.63, 12.14) and 6.01 (5.92, 6.08) among South Asian and non-South Asian men respectively and 5.07 (3.23, 6.91) and 2.99 (2.94, 3.03) among South Asian and non-South Asian women. The incidence rate ratio was 1.45 (95% CI 1.17, 1.78) for South Asian compared to non-South Asian men and 1.80 (95% CI 1.31, 2.48) for South Asian women.

**Table 2:** Incidence rate and number of first admissions for acute MI* in Scotland between 1^st ^May 2001 and 31^st ^December 2003 by sex and ethnic group.

	**Non South Asians**			**South Asians**		
	
	**Rate (Admissions/1000 population/year)**	**Number of cases**	Census population **	**Rate (Admissions/1000 population/year)**	**Number of cases**	Census population **
**Female**						
25 to 34	0.03	29	320,018	0.00	0	3,519
35 to 44	0.20	199	366,337	0.28	2	2,647
45 to 54	0.68	584	322,103	1.36	6	1,653
55 to 64	2.00	1408	264,030	3.49	8	860
65 to 74	5.05	3043	225,973	8.39	10	447
75 to 84	11.33	4605	152,421	24.19	8	124
85 and over	21.92	3406	58,266	42.86	4	35
Crude rate, all ages	2.91	13276	1,709,148	1.53	38	9,285
Standardised rate (95% *CI*), all ages	2.56 (2.51, 2.60)			4.86 (3.05, 6.67)		

**Male**						
25 to 34	0.12	86	278,236	0.00	0	3,328
35 to 44	0.69	609	332,767	1.58	11	2,612
45 to 54	2.36	1948	308,996	2.56	12	1,756
55 to 64	5.43	3525	243,602	6.36	20	1,179
65 to 74	10.28	5091	185,773	15.29	27	662
75 to 84	18.77	4726	94,429	42.55	16	141
85 and over	31.05	1710	20,655	26.79	2	28
Crude rate, all ages	4.53	17696	1,464,458	3.40	88	9,706
Standardised rate (95% *CI*), all ages	5.00 (4.93, 5.08)			7.71 (5.68, 9.75)		

* acute MI defined as ICD10 code I21 in the principal diagnostic position

** restricted to those successfully linked to a Community Health Index record

Between April 2001 and December 2003 there were 17,115 deaths following a first AMI, 40 among South Asians and 17,075 among non-South Asians. Among South Asians 53% (21) of deaths occurred before hospital admission, while among non-South Asians 57% (9,672) of deaths occurred before admission. Table 3 shows that at successive intervals up to 720 days after AMI admission, substantially larger proportions of South Asian men and women than non-South Asians were alive. Age-specific analyses for AMI in the principal position showed that for South Asians the hazard ratios were comparatively low in 35–44 yrs (0.87), 55–64 (0.61), 65–74 (0.62), 75–84 (0.56) and 85+ (0.87) age groups. The only exception was the 45–54 yrs age group (1.19, 4 deaths). For AMI in any position the results were similar. The odds ratio (OR) based on logistic regression for death by 30 days following AMI was 0.43 (95% *CI *0.29, 0.63) for South Asians compared with non-South Asians. These lower odds of death were slightly altered by adjustment for age, sex and deprivation quintile (OR 0.57, 95% *CI *0.37, 0.86).

**Table 3:** Proportion alive at 30, 90, 180, 270, 360 and 720 days after AMI admission, by sex and ethnic group.

		**Non South Asian**		**South Asian**	
		**female**	**male**	**female**	**male**
30 days	alive(%)	47.3	56.4	70.6	72.7
	Total(N)	12,974	17,257	34	88
90 days	alive(%)	42.7	53.1	69.7	70.6
	Total(N)	12,415	16,473	33	85
180 days	alive(%)	39.2	49.9	67.7	69.9
	Total(N)	11,773	15,476	31	83
270 days	alive(%)	36.0	46.3	65.5	67.1
	Total(N)	11,186	14,441	29	76
360 days	alive(%)	32.3	42.4	61.5	62.1
	Total(N)	10,579	13,456	26	66
720 days	alive(%)	14.9	21.8	50.0	44.4
	Total(N)	8,418	9,921	20	45

**Figure 2:**
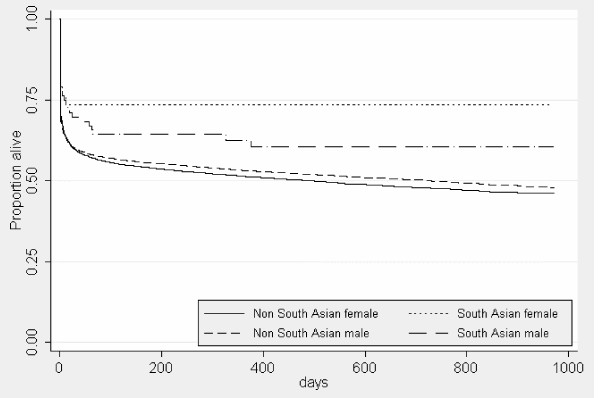
Kaplan-Meier plot of survival following admission for AMI, Scotland 2001–2003, by ethnic group and sex.

An age-adjusted Kaplan-Meier plot of survival by sex and ethnic group is shown in figure 2. In a Cox regression model the hazard ratio among South Asians compared to non-South Asians was 0.50 (95% CI 0.37, 0.68). After adjustment for age, sex and any previous admission for diabetes the hazard ratio was 0.59 (95% CI 0.43, 0.81). With those over 75 years of age excluded (associations between disease and social economic inequalities are least in the elderly) the hazard ratio was 0.68 (95% CI 0.46, 1.01). These results were unaltered when subjected to checking procedures.

## Discussion

### Ethics and methods

We surmounted formidable technical, ethical, administrative and legal challenges to link census, CHI, hospital and mortality data for analysis of the relationship between ethnicity and disease. It is vital to demonstrate confidentiality, and in particular to address the public's concerns. The encryption ensured records could be linked in an unidentifiable form, but our security procedures provided additional safeguards. It is feasible for someone to try out a large number of possible CHI numbers to look for the corresponding encrypted code. This can be avoided by using extra encryption [20].

There is an obligation to ensure that potential ethnic inequalities are highlighted so that they can be assessed and addressed [1,17]. We had to balance individuals' right to data privacy (individual consent was impossible) and the potential benefits to society of producing information derived from potentially sensitive data [21]. We judge our methods struck the balance. The proportion of records ascribed an ethnicity code (94%) was slightly smaller than that normally achieved within ISD (typically around 98%). As the non-White ethnic minority population (about 2%) is comparatively small the false positive rate is critical. A rigorous matching methodology appropriate for administrative matching was, therefore, used. Census forms are completed by the public and processed electronically using optical recognition and keying from images. The success of these processes depends on legibility. The detailed spelling of a name, particularly if it is associated with a minority ethnic group, is more likely to be prone to error when transcribed by a third party onto NHS records, than in the census where the census informant writes it. Such errors could lead to varying accuracy of linkage by ethnic group. Nevertheless, we met our prior stated standard of 80% for every ethnic group. In future, we hope to examine matching rates at each stage of the linkage and by sex.

### Acute myocardial infarction-strengths and weaknesses of the analysis

The major strength of this analysis is the population coverage with the inclusion of community and hospital deaths as the SMR 01 database contains over 99% of hospital admissions for AMI in Scotland (A Redpath, ISD, personal communication, 2006). Possible, as opposed to probable or definite, AMIs are coded as chest pain and are excluded from our figures. The quality of data on AMI in the database has been validated as reliable [19].

The diagnosis of AMI was based on criteria used by clinicians to make the diagnosis, however, which would be inconsistent over time. This should not, however, affect ethnic groups differently. If misclassification of diagnosis is non-differential then the differences would probably be underestimated, but if they were differential differences would be exaggerated. We do not have data to assess these options, and the anonymised methods precluded validity studies at this stage, but are a high priority for future work.

Linkage rates were slightly lower for South Asians than non-South Asians. If non-linkage occurred at random, as is likely, this would reduce the power of the study, but not bias the results. If those not matched were at different risk of AMI or death following AMI, from those who were matched, this would bias the results. We intend to explore this in future research. The limited information available to allow adjustment for co-morbidity did not explain differences in survival, and in future analyses, assuming ethical permission can be obtained, we would include more explanatory variables from the Census.

We did not separate Indians and Pakistanis (the dominant South Asian populations in Scotland), as desirable [11], because of lack of numbers, but this is technically feasible, and is planned for the future as events accrue. The pattern of cardiovascular risk factors varies among different South Asian populations [11], but the excess mortality is shared by all [15].

### Findings on incidence of and survival from acute myocardial infarction in relation to previous knowledge

Few studies have reported the incidence of CHD among South Asians in the UK [15]. Our findings corroborate those of Tunstall-Pedoe and colleagues  who reported a 30% (not statistically significant) increased rate of first and recurrent AMI among Asian (predominantly Bangladeshi) men in London [16]. The data accord with high CHD mortality in South Asian-born populations, compared to White populations [15,22].

Studies of hospital admission rates for AMI in the UK have reported a similar [15,23] or higher [24,25] risk for South Asians, but these did not include community deaths. Wilkinson [26] reported a two-fold and Hughes a 4.9 fold increase in the incidence of AMI [27] – the latter result being an outlier. Britton et al reported a doubling of the incidence of fatal CHD or MI in 560 South Asian civil servants compared to White ones in the Whitehall 2 cohort study [28]. Our findings are consistent with the relatively high CHD mortality in those born in the Indian subcontinent around the 1971–2001 Censuses [1,10,29-31] and the relatively high CHD prevalence [11,12,32].

As survival from AMI was comparatively good, our data indicate that the high rates of CHD mortality arise from higher incidence rather than from higher case fatality. Previous studies of fatality after AMI in South Asians were on hospital cases [33]. Muhktar found no significant difference in survival up to 4 years after discharge in 111 South Asians and matched non-South Asian controls [34]. Studies agreeing with this conclusion include those by Lowry et al in Birmingham [35], Hughes et al in London [27], Lear et al in Leicester [36], Liew et al in London [37], Gupta et al in Toronto, Canada [38], and Mak et al in Singapore [33]. Wilkinson et al's work is unusual in showing a substantially poorer survival in South Asians [26]. Lawrence and Littler's data showed better survival in 124 South Asians in Birmingham compared with matched White people [39]. Unpublished data from the UK national audit (MINAP) show 'Asians' were substantially less likely to die from AMI than 'Caucasians' (Zaman, M. J., lecture, London School of Hygiene and Tropical Medicine, 12/1/07).

Our results of better survival are unlikely to be chance or analytic errors. How might we explain them? Were South Asians diagnosed at an earlier stage of their disease than the rest of the population? With the recent emphasis on heart disease in South Asians this is possible. South Asians may have been quicker to seek medical attention as suggested by Chaturvedi [40]. Although South Asians may present promptly to hospital they are more likely to encounter delays between arrival and treatment as shown in some (but not all) studies in the UK [41] and Canada [38]. South Asians may have quicker access to emergency treatment in Scotland, particularly in Glasgow and Edinburgh (where most of them live), because of the proximity of their inner-city residences to major hospitals. South Asians are less likely to get revascularisation as shown by Feder et al in London [42].

Alternatively, non-South Asians, who are mainly White Scottish people, may have particularly poor survival in Scotland while South Asians in Scotland have average survival. There is evidence for this. Our mortality patterns in South Asians are similar to those reported by Mak et al in Singapore [33] and compatible with the in-hospital mortality figures in studies by Mukhtar et al [34], Lowry et al [35], Lear et al [36], and Gupta et al [38]. Macintyre et al [43] have shown an association between social and economic deprivation and survival in Scotland, which might account for Scotland's relatively high mortality. This still leaves the question of why this might not apply to South Asians. In our results, adjustment for deprivation accounted for some but not all of the difference in survival between South Asians and non-South Asians.

The greater risk of AMI among South Asians but better prognosis needs further research including on the possible occurrence of AMI at an early stage in the progression of atheroma [44]. Anatomical differences in the coronary artery vasculature in South Asians [45] disappear when populations are matched closely, and are unlikely to explain the high risk[46].

## Conclusions: public health and clinical practice and research

It is worrying that South Asians in Scotland are at greater risk of heart attack than a Scottish population internationally notorious for its susceptibility to heart disease. The clinical and epidemiological challenges for prevention, control and rehabilitation of CHD in South Asians are formidable. Fortunately, survival after AMI in South Asians seems to be comparatively good in Scotland, and similar to comparison populations elsewhere. Nonetheless, incidence and mortality needs to be driven even lower through better treatment and prevention.

This project has created a retrospective cohort of about 4.6 million people living in Scotland at the time of the 2001 Census. Subject to ethical approval, future (now funded) work will have the potential to examine health care procedures and adjust for other variables that are available in the census. This new work will focus on cardiovascular disease, cancers, mental health and maternal and child health. At its conclusion, scheduled for the end of 2009, we will be able to provide more detailed guidance on feasibility and costs (currently estimated at £300,000).

Our approach, potentially, has international applicability. It demonstrates how the glaring absence of cohort studies reporting by ethnic group in Europe can be overcome [47]. There is considerable potential in linking databases that have previously been considered too sensitive for record linkage or where linkage is restricted by data protection legislation [48]. The methods described here and in more detail in our report [17] have the potential to fill the information gap on data by ethnic group. This gap will persist until we have high quality prospective ethnic group coding systems in health-care databases and the inclusion of an ethnic code on birth and death registration – both formidable long-term challenges, hitherto unachieved in either Europe or North America [1].

## Competing interests

The author(s) declare that they have no competing interests.

## Authors' contributions

RB was the originator of the project and the principal applicant for the grant with JC and CF as co-applicants. RB and CF set up the project, with full involvement from JC, DK and JJ. CP had the idea of linking the census data to the data held by ISD and he performed most of the linkage work including the development of the methods.

JC had the original plan for the use of one-way encryption and led in drafting the methods components of this paper. GM was the main contributor from GROS, making both practical and intellectual contributions. CF, RB, and MS led the analytic plan for analysis of myocardial infarction and CF and MS did the analysis. RB, CP, GM and CF are guarantors for this paper. All authors contributed to the design of the study, the writing, and read and approved the final version of the manuscript.

## Appendix 1

### What was known before

• There are important ethnic/racial variations in disease and health care

• Prospective ethnic coding is required or recommended in several countries, but has been very difficult to implement

• Record linkage techniques have potential in meeting the need for data

• South Asian populations have high CHD mortality rates, but incidence and survival data are sparse, and not based on population-based cohort analyses

### What is known now?

Ethical and legal obstacles to linking, retrospectively, ethnic codes in the UK census to NHS health and mortality databases are surmountable

• 94% of records can be linked, with more than 80% in every census based ethnic group

• The method creates a retrospective cohort study where ethnic group and other social status data come from the census and health outcomes and procedures come from NHS databases

• The incidence of acute myocardial infarction was substantially higher in South Asians in Scotland than in the remainder of the population

• Survival following acute myocardial infarction was better in South Asians than in the remainder of the population

The method has international implications and mandates review of current policies on prospective coding by ethnic group

## Pre-publication history

The pre-publication history for this paper can be accessed here:


